# Argentine Tango as an Integrative Therapeutic Approach: Bridging Dance Therapy and Melotherapy

**DOI:** 10.3390/jfmk11040408

**Published:** 2026-10-05

**Authors:** Csongor Kicsi

**Affiliations:** Faculty of Physical Therapy, National University of Physical Education and Sport, 060057 Bucharest, Romania; csongor.kicsi@unefs.ro

**Keywords:** Argentine tango, dance therapy, melotherapy, music therapy, rehabilitation, healthy aging, neurological rehabilitation, emotional regulation, rhythmic stimulation, interdisciplinary approaches

## Abstract

Argentine tango integrates rhythmic auditory information, adaptive locomotion, improvisation, and continuous partnered interaction within a complex dance practice. This review examines its therapeutic relevance with primary emphasis on neurological rehabilitation, particularly Parkinson’s disease (PD), where tango-specific evidence is most developed. Particular attention is given to balance, functional mobility, gait adaptability, and motor–cognitive integration, while cognitive, emotional, and social outcomes are considered complementary dimensions rather than equivalent primary endpoints. This review examines mechanisms involving auditory–motor coupling, motor learning, embodied cognition, and interpersonal coordination, while distinguishing tango-based interventions from formal dance/movement therapy and music therapy. Particular consideration is given to Adapted Tango, including how task demands can be scaled while preserving defining characteristics of Argentine tango. Rather than claiming superiority over other dance forms, the review proposes that tango is therapeutically relevant because of its distinctive combination of walking-based multidirectional locomotion, musical interpretation, improvisational decision-making, and partner-derived sensory information. These relationships are integrated within the Argentine Tango Therapeutic Integration Model (ATTIM), proposed as a hypothesis-generating framework for future research.

## 1. Introduction

Dance is a complex form of human expression in which movement, music, emotion, cognition, and social interaction converge within an embodied experience. Across cultures and historical periods, dancing has served artistic, ritual, recreational, communicative, and social functions, while contemporary research increasingly recognizes its potential relevance to health and well-being. Dance-based interventions engage multiple domains simultaneously, requiring motor coordination, sensory integration, attention, memory, affective processing, and interpersonal adaptation. Accordingly, evidence from dance and dance movement therapy research suggests beneficial effects across psychological, cognitive, interpersonal, and psychomotor domains, although substantial heterogeneity exists among interventions and populations [1].

Argentine tango represents a particularly compelling example of this multidimensionality. As an artistic and social practice, it is characterized by partnered interaction, improvisation, continuous adaptation, and the close relationship between movement and musical structure. Unlike predominantly choreographed dance forms, social Argentine tango is fundamentally improvisational: dancers operate within a shared movement vocabulary while continuously negotiating direction, timing, spatial organization, and expressive intention [2].

Music is therefore not merely an accompaniment to movement but an organizing component of the dancing experience, while the partner becomes an additional source of sensory, motor, affective, and communicative information.

These characteristics have increasingly attracted attention within rehabilitation and health sciences. Pioneering empirical work by Madeleine E. Hackney and colleagues demonstrated that Argentine tango could be adapted for individuals with Parkinson’s disease and investigated systematically as a therapeutic intervention. Studies reported improvements in outcomes related to balance, functional mobility, gait, and quality of life, while comparisons with other forms of partnered dance suggested that specific characteristics of tango may be particularly relevant to motor deficits associated with Parkinson’s disease [3,4].

More recent work has broadened this perspective toward Adapted Tango and its potential application to motor and cognitive functioning in neurodegenerative conditions [5]. Importantly, however, the therapeutic relevance of tango cannot be reduced to locomotor exercise alone. Research has also highlighted embodiment, aesthetic experience, body self-efficacy, interpersonal connection, enjoyment, and psychological well-being as potentially meaningful dimensions of tango-based interventions [6].

This multidimensional character becomes particularly relevant when tango is considered at the intersection of dance therapy and melotherapy (music therapy). Movement-based therapeutic approaches emphasize embodiment, bodily expression, sensorimotor experience, and psychosocial integration, whereas music-based approaches exploit rhythm, musical structure, affective engagement, and auditory–motor relationships. Argentine tango naturally combines elements belonging to both domains: rhythmic and melodic information is transformed into movement; movement is continuously adjusted through proprioceptive and tactile information; and both processes occur within an interpersonal relationship. Rather than treating music, movement, and social interaction as independent therapeutic components, tango provides a context in which they operate concurrently and reciprocally.

The primary aim of this narrative and integrative review is to synthesize the available evidence on the therapeutic application of Argentine tango, with particular emphasis on Parkinson’s disease and motor and motor–cognitive outcomes, where tango-specific evidence is most developed. A secondary aim is to examine plausible mechanisms that may help explain these effects by integrating three explicitly differentiated bodies of evidence: direct evidence from Argentine tango interventions; related evidence from dance and dance/movement-based interventions; and indirect mechanistic evidence from research on rhythm, auditory–motor coupling, music-based interventions, motor learning, and interpersonal synchronization. On this basis, the review develops the Argentine Tango Therapeutic Integration Model (ATTIM) as an author-generated, hypothesis-generating conceptual framework intended to organize existing evidence and identify testable relationships rather than to represent an empirically validated therapeutic model.

ATTIM conceptualizes these processes across four interconnected levels: (I) therapeutic inputs, comprising music, movement, and partner interaction; (II) neuropsychological and relational mechanisms, including auditory–motor coupling, embodied cognition, emotional regulation, motor learning and neuroplasticity, and interpersonal synchrony; (III) therapeutic outcomes, encompassing motor function, cognitive performance, emotional well-being, social participation, and quality of life; and (IV) contextual and clinical moderators, comprising participant-, intervention-, provider-, and environment-related factors that may influence the strength and direction of these therapeutic pathways. Rather than proposing a simple linear causal sequence, ATTIM conceptualizes Argentine tango as a dynamic therapeutic system in which these levels interact continuously and reciprocally. The framework thus provides a foundation for interpreting existing evidence and generating testable hypotheses for future interdisciplinary research.

## 2. Review Methodology

### 2.1. Review Design and Scope

This study was designed as a narrative and integrative review with conceptual synthesis, rather than a systematic review or meta-analysis. Its primary focus is the therapeutic application of Argentine tango in neurological rehabilitation, particularly Parkinson’s disease (PD), where tango-specific evidence is most developed. Motor and motor–cognitive outcomes therefore receive the greatest emphasis, whereas evidence concerning healthy aging, cognition, emotional well-being, social participation, and quality of life is considered complementary or emerging.

To distinguish the strength and directness of the evidence, sources were classified as: direct evidence, derived from Argentine or Adapted Tango interventions; related evidence, derived from other dance or dance/movement-based interventions; and indirect mechanistic evidence, addressing potentially relevant mechanisms such as rhythmic auditory stimulation, auditory–motor coupling, motor learning, neuroplasticity, embodied cognition, and interpersonal synchronization.

ATTIM was developed as an author-generated, hypothesis-generating conceptual framework intended to organize these bodies of evidence rather than as an empirically validated causal model.

### 2.2. Information Sources and Search Strategy

The initial literature base was developed iteratively during preparation of the narrative review. A structured supplementary search was subsequently conducted to improve transparency and verify the tango-specific evidence base. PubMed/MEDLINE was searched from database inception to 27 September 2026.

The principal search combined terms identifying Argentine tango and its adapted forms:

(“Argentine tango”[Title/Abstract] OR “Argentinian tango”[Title/Abstract] OR “adapted tango”[Title/Abstract] OR “tango dancing”[Title/Abstract] OR “tango dance”[Title/Abstract])

A clinically focused search was additionally conducted using the following query:

((“Argentine tango”[Title/Abstract] OR “Argentinian tango”[Title/Abstract] OR “adapted tango”[Title/Abstract] OR “tango dancing”[Title/Abstract] OR “tango dance”[Title/Abstract]) AND (Parkinson[Title/Abstract] OR rehabilitation[Title/Abstract] OR therap[Title/Abstract] OR gait[Title/Abstract] OR balance[Title/Abstract] OR mobility[Title/Abstract] OR cognition[Title/Abstract] OR aging[Title/Abstract] OR “older adult”[Title/Abstract] OR “older adults”[Title/Abstract]))

Reference lists of relevant tango-specific reviews and primary studies were also examined to identify additional eligible publications.

The literature addressing dance, music-based rehabilitation, rhythmic auditory stimulation, neuroplasticity, and interpersonal synchronization was selected separately when required to interpret proposed mechanisms. These sources were used to support mechanistic plausibility and were not treated as direct evidence of tango-specific therapeutic effects.

ChatGPT (OpenAI, GPT-5.6 Sol, San Francisco, CA, USA; https://chatgpt.com/; accessed on 8 September 2026 was used as an auxiliary tool for literature discovery, organization, and conceptual structuring, but not as a bibliographic database or autonomous study-selection system. All references considered for inclusion were verified by the author against their original bibliographic records or publications.

### 2.3. Eligibility and Study Selection

For the direct clinical evidence synthesis, peer-reviewed human studies were eligible when Argentine Tango or Adapted Tango constituted an identifiable intervention, and at least one motor, functional, cognitive, psychological, participation-related, feasibility, safety, adherence, or biological outcome was reported. Randomized and non-randomized controlled studies, prospective and pre–post interventions, pilot and feasibility studies, and qualitative investigations were considered. Tango-specific systematic reviews and meta-analyses were retained as secondary evidence syntheses.

Studies were excluded from the direct evidence synthesis when Argentine tango was not an identifiable intervention, when tango-specific effects could not be distinguished from a broader intervention, or when the publication contained no relevant human clinical or functional outcomes. Studies of other dance forms, music therapy, rhythmic auditory stimulation, or related neuropsychological mechanisms were classified separately as related or indirect evidence rather than tango-specific evidence.

Study selection was performed by the sole author, who screened titles and abstracts and subsequently examined potentially eligible publications. AI-assisted tools did not independently determine eligibility.

Because this article was designed as a narrative and integrative review rather than a systematic effectiveness review, no formal risk-of-bias instrument was applied across all included studies. Methodological limitations reported in individual studies and tango-specific systematic reviews were nevertheless considered when interpreting the evidence.

### 2.4. Data Extraction and Evidence Classification

For tango-specific intervention studies, information was extracted on study design, sample characteristics, population, intervention characteristics, intervention duration, comparator, assessed outcomes, measurement instruments, principal findings, and reported methodological limitations.

To prevent indirect evidence from being interpreted as tango-specific evidence, sources were classified into three categories. Direct evidence comprised primary studies in which Argentine or Adapted Tango was the intervention under investigation. Tango-specific systematic reviews and meta-analyses were considered separately as secondary evidence syntheses. Related evidence comprised studies and reviews examining other forms of dance, dance/movement interventions, or broader dance-related health effects. Indirect mechanistic evidence comprised studies addressing mechanisms relevant to the theoretical interpretation of tango, including rhythmic auditory stimulation, auditory–motor coupling, music-based rehabilitation, motor learning, neuroplasticity, and interpersonal synchronization. Definitional and cultural sources were used only for conceptual or disciplinary context and were not treated as evidence of therapeutic efficacy.

### 2.5. Conceptual Synthesis and Development of ATTIM

ATTIM was developed as an author-generated conceptual synthesis rather than as the outcome of a statistical model or formal inductive qualitative analysis. The framework was constructed by integrating recurrent concepts identified across the three evidence categories while maintaining their different evidentiary status. Tango-specific structural characteristics informed Level I (therapeutic inputs); direct clinical findings and supporting mechanistic literature informed the proposed relationships represented in Levels II and III; and recurrent participant-, intervention-, provider-, and environment-related considerations informed Level IV.

Importantly, direct tango-specific evidence was given priority when characterizing therapeutic outcomes, whereas related and indirect evidence was used primarily to support mechanistic plausibility. The relationships represented within ATTIM therefore constitute theoretically informed hypotheses rather than established causal pathways. The model is intended to organize existing evidence, make evidentiary gaps explicit, and generate testable predictions for subsequent empirical investigation.

## 3. Argentine Tango as a Multidimensional Practice

### 3.1. Historical and Sociocultural Foundations

Argentine tango emerged in the late nineteenth century within the rapidly transforming urban environment of the Río de la Plata, particularly Buenos Aires and Montevideo. Its development cannot be attributed to a single cultural tradition; rather, tango arose through interactions among European immigrants, Afro-Rioplatense communities, criollo populations, and other groups inhabiting these expanding port cities. This cultural hybridity contributed to the formation of an artistic expression combining music, dance, poetry, and social practices. In recognition of this significance, UNESCO inscribed tango on the Representative List of the Intangible Cultural Heritage of Humanity in 2009, emphasizing both its Argentine and Uruguayan heritage and its role in cultural dialogue [7].

Tango subsequently evolved from a localized urban practice into an internationally disseminated cultural phenomenon. Importantly, this globalization did not eliminate its social character. The *milonga*—both a musical genre associated with tango and the social environment in which tango is danced—remains a space structured by interpersonal encounters, shared conventions, observation, invitation, and collective participation. Thus, Argentine tango extends beyond the execution of dance movements: it constitutes an embodied social practice through which individuals negotiate identity, proximity, communication, and belonging. These characteristics are particularly relevant when considering tango in relation to well-being, because its potential effects arise within a culturally meaningful activity rather than an isolated exercise protocol.

### 3.2. Music, Rhythm, Phrasing, and Emotional Expression

Music is inseparable from the embodied organization of Argentine tango. Tango music provides temporal structure while simultaneously inviting interpretative variability. Dancers respond not only to an underlying pulse but also to rhythmic accents, pauses, melodic contours, changes in intensity, and larger musical phrases. Consequently, the relationship between music and movement is not limited to synchronization with a repetitive beat; dancers continuously select how musical information will be embodied.

This distinction is therapeutically relevant. Rhythmic information can provide external temporal organization for movement, whereas variations in tempo and phrasing require anticipation and adaptation. Tango therefore combines rhythmic predictability with controlled variability. In neurological populations, external rhythmic structure may facilitate movement organization, while the need to respond flexibly to musical changes potentially increases attentional and motor-planning demands [8].

Musical interpretation also provides tango with an affective dimension. Changes in melody, dynamics, articulation, and phrasing may influence movement quality and expressive intention. The dancer does not merely hear the music but translates perceived musical properties into timing, direction, amplitude, pauses, and interpersonal movement. Tango can therefore be conceptualized as a form of embodied musical interpretation, a concept that later becomes important for understanding its position between dance-based and music-mediated therapeutic approaches.

### 3.3. Walking, Embrace, Improvisation, and Partner Communication

The fundamental motor vocabulary of Argentine tango is closely related to walking. However, tango walking transforms ordinary locomotion into a complex sensorimotor task involving controlled weight transfer, initiation and termination of movement, changes in direction, variable step length, backward and lateral stepping, pivoting, and continuous postural adaptation. These characteristics overlap with motor domains commonly targeted in rehabilitation, particularly dynamic balance, gait adaptability, anticipatory postural control, and coordination.

A second defining feature is the *abrazo*, or embrace. Through the dance connection, partners exchange continuous tactile, proprioceptive, kinesthetic, and mechanical information. Partner communication is therefore predominantly non-verbal and embodied. Rather than executing two independent choreographies simultaneously, dancers continuously regulate their actions relative to each other.

This relationship is especially evident in improvisation. Argentine tango is not fundamentally organized around reproducing a fixed sequence of steps. Instead, movement emerges through moment-to-moment decisions constrained by musical structure, available space, movement possibilities, and information transmitted between partners. Improvisation therefore requires attention, movement selection, anticipation, inhibition, spatial processing, and rapid sensorimotor adaptation.

These features distinguish tango from exercise based primarily on repetitive motor execution. The dancer must simultaneously process information originating from the music, the partner, the environment, and the body itself. Partnering may additionally contribute motivational and social dimensions. In individuals with Parkinson’s disease, both partnered and non-partnered tango have been associated with improvements in gait and balance, while participants in partnered classes reported greater enjoyment and interest in continuing the activity [9]. Thus, the embrace should not be interpreted simply as physical support; it constitutes an interpersonal information channel and an important component of the tango experience.

### 3.4. Why Argentine Tango? Distinctive Task Architecture and Comparison with Other Dance Forms

The therapeutic rationale for Argentine tango should not be interpreted as evidence that tango is universally superior to other forms of dance. Ballroom dances, culturally embedded dances, and other structured or improvisational movement practices may engage many of the same physical, cognitive, affective, and social processes. The relevance of Argentine tango lies instead in the particular configuration of demands that are integrated continuously during the dance.

Argentine tango combines walking-based locomotion with frequent movement initiation and termination, forward, backward, and lateral stepping, variable step length and velocity, turning, dynamic weight transfer, controlled pauses, and continuous postural adaptation. At the same time, movement sequences are not primarily reproduced from fixed choreography but generated online through improvisational decisions constrained by musical structure, partner-derived information, available space, and the dancer’s current postural state. Partner communication provides an additional stream of tactile and proprioceptive information that continuously modifies subsequent movement. Tango therefore combines gait adaptability, musical interpretation, online decision-making, and reciprocal sensorimotor communication within the same task.

This configuration may be particularly relevant to Parkinson’s disease, in which difficulties with internally generated movement, movement initiation, step scaling, turning, postural control, and cognitive–motor integration can contribute to mobility limitations. Importantly, however, this rationale does not establish that tango is inherently more therapeutic than other dance modalities. Hackney and Earhart [3] directly compared Argentine tango with American ballroom dancing in individuals with PD and found beneficial effects associated with both approaches, while suggesting differential effects on selected movement outcomes. Such findings support further investigation of tango-specific task characteristics but do not justify a general claim of superiority.

Other dance forms may provide overlapping or alternative combinations of rhythmic, motor, cognitive, expressive, cultural, and interpersonal demands. The clinically relevant question is therefore not whether Argentine tango is universally “better” than ballroom or culturally specific dances, but whether its particular task architecture is especially well matched to defined therapeutic targets. Direct comparative and component-based studies are required to determine which benefits reflect dance participation generally and which, if any, depend specifically on characteristics of Argentine tango.

### 3.5. From Social Dance to Therapeutic Intervention

The transition of Argentine tango from social practice to therapeutic intervention did not require the invention of an entirely new movement system. Rather, researchers recognized that core characteristics of tango—including rhythmic cueing, adaptable gait, dynamic postural control, and partnered improvisation—overlap with important rehabilitation targets.

Pioneering research by Hackney and colleagues provided empirical support for this translation. An early controlled study demonstrated improvements in functional mobility among individuals with Parkinson’s disease following tango training [10]. Subsequent studies examined intensive tango programs and comparisons with other ballroom dances, reporting improvements in selected measures of balance, gait, and motor function [3,11]. Later systematic synthesis found favorable effects particularly for motor severity, balance, and functional mobility, although small samples and methodological heterogeneity limit definitive conclusions [8].

The therapeutic relevance of Argentine tango may therefore derive precisely from its multidimensional nature. Music provides temporal and affective information; walking and improvisation create adaptable motor challenges; the embrace establishes an embodied interpersonal relationship; and the social context provides meaning, enjoyment, and participation. These components should not be considered independent therapeutic ingredients. They continuously interact during the dance experience. This convergence provides the conceptual foundation for considering Argentine tango not merely as physical exercise accompanied by music, but as an integrative practice situated at the intersection of movement, musical experience, cognition, emotion, and human interaction.

## 4. Dance Therapy and Melotherapy: Conceptual Foundations

The evidence discussed in this section is primarily conceptual, definitional, and related evidence and should not be interpreted as direct evidence of Argentine tango-specific therapeutic effects.

### 4.1. Dance Therapy and Dance/Movement Therapy

The therapeutic use of dance is grounded in the broader recognition that bodily movement can influence physical, psychological, cognitive, and interpersonal functioning. Nevertheless, an important distinction must be made between dance as a health-promoting intervention and dance/movement therapy (DMT) as a professional therapeutic discipline. The American Dance Therapy Association defines DMT as the psychotherapeutic use of movement to promote the emotional, social, cognitive, and physical integration of the individual, with the purpose of improving health and well-being [12]. DMT therefore extends beyond participation in structured dance activity: movement functions simultaneously as a means of expression, communication, assessment, and therapeutic intervention within a therapeutic relationship.

The conceptual basis of DMT is strongly associated with embodiment and the reciprocal relationship between bodily experience and psychological processes. Movement may provide access to experiences that are difficult to articulate verbally, while improvisation, mirroring, body awareness, symbolic movement, and interpersonal interaction may facilitate emotional expression, agency, self-perception, and relational engagement. From this perspective, the body is not simply the biomechanical instrument through which therapy is performed; bodily experience is itself part of the therapeutic process.

Evidence supports a distinction between formal DMT and broader dance interventions. In a meta-analysis of 41 controlled studies involving 2374 participants, Koch et al. [1] found an overall moderate effect of DMT and dance interventions on health-related outcomes, although considerable heterogeneity was present. DMT showed smaller but more homogeneous effects, particularly for psychological and cognitive outcomes, whereas general dance interventions demonstrated larger but more heterogeneous effects and appeared especially relevant to psychomotor outcomes. These findings reinforce the importance of avoiding conceptual equivalence between therapeutic dance participation and professionally delivered DMT.

### 4.2. Melotherapy and Music Therapy

The term melotherapy, derived from the Greek *melos* (melody or song) and *therapeia* (treatment or care), has historically been used to describe therapeutic applications of music. In contemporary international clinical and scientific discourse, however, music therapy is the standardized professional term. Accordingly, in this review, melotherapy is retained to emphasize the therapeutic role of music while being discussed in relation to the established discipline of music therapy.

The World Federation of Music Therapy [13] defines music therapy as a professional discipline using music-based experiences—including listening, singing, instrumental performance, and improvisation—to promote health, well-being, capacity, and development at personal and social levels. Importantly, music therapy is delivered by trained professionals within a therapeutic relationship and is grounded in research, professional education, clinical training, and ethical practice. Thus, as with DMT, exposure to music or participation in a music-based activity should not automatically be classified as music therapy.

Music nevertheless possesses characteristics with substantial therapeutic relevance. Rhythm provides temporal structure; melody and harmony contribute to affective processing; musical expectation recruits attention and prediction; and active music-making combines sensory, motor, cognitive, and emotional processes. Particularly relevant to rehabilitation is auditory–motor entrainment, whereby temporal regularities in auditory stimuli can influence the timing and organization of movement. Research on rhythmic auditory stimulation has demonstrated that rhythm can provide an external temporal framework for motor control and has contributed substantially to the development of neurologic music therapy [14].

Consequently, music can operate simultaneously as a temporal cue, motivational stimulus, emotional medium, and framework for sensorimotor organization. These properties are especially relevant to Argentine tango because its music is not incidental background stimulation: dancers continuously transform musical timing, accents, phrases, and expressive characteristics into movement.

### 4.3. Shared Mechanisms and Disciplinary Distinctions

Dance-based and music-based therapies remain distinct disciplines, with different professional identities, theoretical traditions, training requirements, and therapeutic procedures. Nevertheless, they share several mechanisms capable of influencing health and well-being. Both can involve rhythm, attention, prediction, learning, emotional expression, reward, creativity, agency, social interaction, and non-verbal communication. When movement and music occur concurrently, these mechanisms may interact rather than operate independently.

Koch [15] proposed several clusters of mechanisms relevant to creative arts therapies, including pleasure and play, aesthetic experience, non-verbal meaning-making, agency and self-efficacy, and creative generation. Dance additionally provides physical activity, sensorimotor stimulation, proprioceptive feedback, postural adaptation, and spatial exploration. Music contributes temporal organization, auditory prediction, affective modulation, and auditory–motor coupling. Both may also facilitate social cohesion and interpersonal engagement when practiced collectively.

Their therapeutic targets and methods, however, should not be conflated. DMT primarily conceptualizes movement and embodied interaction within a psychotherapeutic process, whereas music therapy uses music-based experiences within a professionally structured therapeutic relationship. Neurologic music therapy further applies evidence-based music and rhythm techniques to cognitive, sensory, speech-language, and motor rehabilitation [16]. These disciplinary boundaries are particularly important when considering tango: a tango class may produce therapeutic benefits without constituting either formal DMT or music therapy.

### 4.4. Argentine Tango at the Intersection of Music and Movement

Argentine tango provides an unusual context in which the principal modalities emphasized by these disciplines become structurally inseparable. The dancer listens, predicts, selects, moves, adapts, communicates, and expresses within a continuous perception–action cycle. Musical rhythm and phrasing influence movement timing; movement alters the sensory information available to both partners; tactile and proprioceptive communication modifies subsequent motor decisions; and emotional interpretation can influence movement quality and interpersonal interaction. Tango can therefore be understood as embodied musical interpretation enacted through interpersonal movement.

This integration is particularly apparent in improvisation. Unlike the reproduction of a predetermined sequence, social tango requires dancers to continuously generate movement solutions in response to music, partner-derived information, spatial constraints, and their own postural state. Such demands combine auditory processing with motor planning, balance, attention, prediction, executive control, and social communication. The relevance of these characteristics to rehabilitation is supported by studies demonstrating improvements in balance, gait, and functional mobility following tango interventions in individuals with Parkinson’s disease [3,8].

Argentine tango should nevertheless not be described simply as “dance therapy” or “music therapy in motion”. Such terminology would obscure important professional and conceptual distinctions. A more defensible interpretation is that tango represents a multimodal therapeutic practice capable of engaging mechanisms relevant to both dance-based and music-based therapies, while adding a distinctive third component: continuous embodied interaction with a partner.

This intersection provides the theoretical foundation for the integrative perspective developed in this review. Music, movement, and partner interaction can be regarded as complementary therapeutic inputs whose simultaneous engagement may generate interconnected sensorimotor, cognitive, emotional, and relational processes. These principles form the conceptual basis of the Argentine Tango Therapeutic Integration Model (ATTIM) developed later in the review.

## 5. Therapeutic Mechanisms of Argentine Tango

The following section distinguishes between mechanisms directly investigated within tango interventions and mechanisms inferred from related dance or indirect mechanistic literature. Where tango-specific mechanistic evidence is unavailable, this limitation is stated explicitly.

The therapeutic potential of Argentine tango is unlikely to depend on a single physiological or psychological mechanism. Rather, tango simultaneously engages auditory, motor, cognitive, affective, and interpersonal systems within a continuously changing sensorimotor environment. This multimodal organization is particularly relevant to rehabilitation because therapeutic outcomes may emerge from interactions among these processes rather than from isolated effects of physical activity, music, or social participation. The following mechanisms provide the principal theoretical basis for understanding this integration. Given the distribution of the current evidence, the following discussion gives greatest clinical weight to motor and motor–cognitive mechanisms relevant to PD; affective and interpersonal processes are considered primarily as complementary mechanisms that may influence engagement, adherence, and participation rather than as independently established therapeutic effects.

### 5.1. Rhythmic Auditory Stimulation and Auditory–Motor Coupling

Human movement is highly responsive to temporal regularities in sound. Auditory–motor entrainment describes the capacity of rhythmic auditory information to influence the timing and organization of movement through interactions between auditory and motor networks. Rhythmic auditory stimulation (RAS) has consequently become an established approach in neurologic rehabilitation, particularly for gait disorders. In Parkinson’s disease (PD), external auditory rhythm may provide temporal information that partially compensates for impaired internal movement timing associated with basal ganglia dysfunction [14,17]. Meta-analytic evidence indicates that RAS can improve selected gait parameters, particularly gait velocity, step length, and stride length [18,19].

Argentine tango incorporates rhythmic cueing intrinsically, but its musical environment is considerably richer than a metronomic stimulus. Dancers respond to pulse, rhythmic accents, tempo, pauses, melodic contours, and phrase boundaries while deciding when and how these features should be embodied. Tango therefore combines entrainment with interpretation. Movement may coincide with the dominant beat, subdivide it, suspend temporarily during a pause, or follow melodic rather than strictly metric information.

This distinction suggests that tango may engage auditory–motor coupling at multiple levels. Predictable temporal information can facilitate movement organization, whereas variations in musical structure demand anticipation, selection, and adaptation. However, the mechanisms demonstrated for formal RAS should not be assumed to operate identically during tango. Direct neurophysiological investigation of auditory–motor entrainment during Argentine tango remains limited. Thus, RAS research provides a mechanistic foundation rather than definitive evidence of tango-specific neural effects.

### 5.2. Gait, Balance, Postural Control, and Motor Learning

Argentine tango places substantial demands on locomotor and postural control. Its movement vocabulary incorporates forward, backward, and lateral stepping; variable step length; changes in direction and velocity; pivots; weight transfers; movement initiation and termination; and transitions between dynamic movement and controlled stillness. These tasks continuously alter the relationship between the center of mass and base of support and require integration of visual, vestibular, proprioceptive, tactile, and auditory information.

Such characteristics are particularly relevant to PD, in which gait hypokinesia, impaired postural responses, reduced movement amplitude, freezing, and difficulties with internally generated movement sequences may restrict mobility. Tango provides repeated practice of adaptable rather than exclusively stereotyped gait. The dancer must modify stepping according to music, space, and partner-derived information, potentially training gait adaptability alongside balance.

Available clinical evidence supports this motor rationale. A systematic review and meta-analysis found favorable effects of tango on motor severity, balance, and functional mobility in PD, although effects were not consistent across all gait outcomes and evidence for freezing of gait remained inconclusive [8].

Tango may additionally promote motor learning through repeated practice, external feedback, task variability, and progressive adaptation. Rather than reproducing identical movements, dancers repeatedly solve related motor problems under changing constraints. This variability may support flexible movement strategies and sensorimotor recalibration. Nevertheless, claims concerning tango-induced neuroplasticity should remain cautious: behavioral improvements are better established than direct evidence demonstrating tango-specific neural plastic changes.

### 5.3. Attention, Executive Function, Memory, and Dual-Task Performance

Tango is simultaneously a motor and cognitive activity. Improvisational dancing requires continuous allocation of attention to bodily position, movement intention, partner information, music, and environmental space. Movement decisions must be generated and updated rapidly, while inappropriate responses must be inhibited. Consequently, tango potentially engages selective and divided attention, working memory, response inhibition, cognitive flexibility, visuospatial processing, prediction, and executive control.

Improvisation is especially relevant. Unlike fixed choreography, Argentine tango requires dancers to select movements from an available repertoire and organize them contextually. Previously learned motor patterns must therefore be retrieved without becoming rigid sequences. This interaction between memory and online decision-making may impose cognitive demands that differ from repetitive exercise.

Partnered tango also creates conditions resembling complex dual- or multi-task performance. A dancer may simultaneously maintain postural stability, regulate step timing, process auditory information, interpret tactile signals, monitor surrounding couples, and plan the next movement. For individuals with neurological impairment, these competing demands could initially increase task difficulty; when appropriately graded, however, they may provide ecologically relevant practice of cognitive–motor integration.

Evidence for cognitive improvement specifically attributable to Argentine tango is less robust than evidence for motor outcomes. Accordingly, attention, executive function, memory, and dual-task performance should currently be considered important candidate mechanisms and therapeutic targets, rather than established tango-specific effects. This distinction is critical for avoiding mechanistic overinterpretation.

### 5.4. Emotional Regulation, Reward, and Motivation

The therapeutic experience of tango cannot be understood exclusively through motor and cognitive demands. Music and dance engage affective and reward-related processes, and their combination may generate pleasure, aesthetic experience, emotional expression, and intrinsic motivation. Contemporary neuroscience indicates substantial overlap between neural systems involved in music, movement, action, emotion, and reward, although the neural dynamics of real-world interactive dance remain incompletely understood [20].

Tango music provides an emotionally expressive environment, while movement allows this affective information to be embodied. Changes in intensity, phrasing, tempo, and melodic contour can influence movement timing and quality. The resulting experience may facilitate emotional expression without requiring explicit verbalization.

Reward and enjoyment may also have indirect therapeutic importance through adherence. Rehabilitation requires repetition, yet repetitive therapeutic exercise may be difficult to sustain over prolonged periods. An activity perceived as meaningful, pleasurable, socially engaging, and progressively challenging may increase willingness to participate. Reviews of tango interventions in PD have generally reported acceptable adherence and attrition, supporting its feasibility as an adaptable intervention [21]. Motivation should therefore not be regarded merely as a secondary psychological benefit; it may influence therapeutic exposure itself and consequently moderate longer-term outcomes.

### 5.5. Touch, Interpersonal Synchrony, and Social Connectedness

Partner interaction distinguishes Argentine tango from many individual exercise and music-based interventions. The embrace creates a continuous channel for tactile and proprioceptive communication through which dancers perceive changes in orientation, weight transfer, direction, timing, and movement intention. Touch therefore serves both biomechanical and communicative functions.

Successful partnered dancing also requires interpersonal synchrony: the temporal coordination of actions between individuals. Importantly, tango synchrony is not simple simultaneous repetition. Partners occupy complementary roles and continuously adapt to one another. Movement therefore emerges through reciprocal perception and action, making the dyad itself a functional component of the task.

These interactions may have psychosocial implications. Coordinated movement can foster affiliation, cooperation, and a sense of shared experience, while the social dance environment provides opportunities for communication, belonging, and participation. Such dimensions are particularly relevant in chronic neurological conditions, where reduced mobility may contribute to social withdrawal. In tango interventions, social participation may therefore constitute both an outcome and a mechanism capable of influencing motivation, emotional well-being, and adherence.

Nevertheless, this domain requires particular scientific caution. The social and collective neuroscience of dance remains substantially less developed than research on individual movement, and direct evidence connecting tango-specific interpersonal synchrony with clinical outcomes is scarce. Touch, synchrony, and social connectedness should therefore be considered theoretically well-supported but incompletely tested components of tango’s therapeutic action.

Taken together, these mechanisms suggest that Argentine tango functions as a coupled therapeutic system rather than a collection of independent stimuli. Rhythm structures movement; movement generates sensorimotor challenges; improvisation recruits cognitive control; music and successful interaction engage affect and reward; and the partner introduces reciprocal tactile, motor, emotional, and social information. These processes can reinforce one another dynamically. This mechanistic interdependence provides the principal rationale for the Argentine Tango Therapeutic Integration Model (ATTIM), in which music, movement, and partner interaction are conceptualized as therapeutic inputs that act through interconnected neuropsychological and relational mechanisms to influence motor, cognitive, emotional, and social outcomes.

## 6. Empirical Evidence for Argentine Tango-Based Interventions: Primary Focus on Parkinson’s Disease

Table 1 summarizes the principal tango-specific primary studies retained in the clinical evidence synthesis. Particular attention is given to study design, intervention dose, comparator conditions, outcome measures, principal findings, and methodological limitations. Several publications originate from overlapping research cohorts; these reports were retained when they addressed distinct outcomes or follow-up periods, but their shared sample origin was considered when interpreting the apparent volume of evidence.

### 6.1. The Pioneering Work of Madeleine E. Hackney

The clinical investigation of Argentine tango as a rehabilitation strategy has been shaped substantially by the pioneering work of Madeleine E. Hackney and collaborators. Her early studies moved tango from anecdotal and recreational contexts toward systematically investigated therapeutic application, particularly in Parkinson’s disease (PD). Importantly, this research did not conceptualize tango merely as aerobic exercise but examined characteristics directly relevant to neurological rehabilitation, including multidirectional stepping, balance, gait initiation, movement adaptation, partnering, and cognitive–motor engagement.

In an early randomized preliminary study, Hackney et al. [10] compared 20 tango classes with group exercise in 19 individuals with PD. Both interventions improved overall Unified Parkinson’s Disease Rating Scale (UPDRS) scores, whereas significant improvement in Berg Balance Scale performance occurred in the tango group but not the exercise group. Although the small sample precluded strong conclusions, this study provided an important empirical foundation for subsequent tango-based rehabilitation research.

Hackney and Earhart [3] subsequently compared Argentine tango directly with American ballroom dancing. Both dance interventions were associated with beneficial changes, while between-intervention differences on selected outcomes suggested that the movement demands of tango may warrant particular investigation in PD. Importantly, this comparison should not be interpreted as evidence that Argentine tango is universally superior to ballroom dancing; rather, it suggests that different dance forms may impose different motor and sensorimotor demands and therefore produce outcome-specific responses. A complementary intensive intervention showed that even a short-duration program comprising ten 90-min tango sessions over two weeks was associated with improvements in balance and motor severity [11]. Research comparing partnered and non-partnered tango further demonstrated that both formats could improve aspects of balance and gait, while partnered participants expressed greater enjoyment and interest in continuing participation [9].

This research program was later extended beyond conventional motor outcomes. McKee and Hackney [24] investigated spatial cognition while controlling for social interaction through an education comparison condition, thereby advancing the hypothesis that tango may influence cognitive–motor processes rather than mobility alone. Collectively, Hackney’s work established Adapted Tango as the earliest and most extensively investigated tango intervention for PD, providing the methodological and conceptual basis for much of the subsequent literature [21].

### 6.2. Mobility, Gait, and Balance in Parkinson’s Disease

Motor outcomes constitute the strongest empirical domain supporting tango-based interventions. This is theoretically plausible because tango repeatedly challenges gait initiation, termination, backward and lateral stepping, turning, weight transfer, dynamic balance, and adaptation of step timing and amplitude.

The systematic review and meta-analysis by Lötzke et al. [8], incorporating 13 studies, including nine randomized controlled trials, reported significant effects favoring Argentine tango for motor severity measured with the UPDRS-III (effect size [ES] = −0.62), balance measured with the Mini-BESTest (ES = 0.96) and Berg Balance Scale (ES = 0.45), and functional mobility measured with the Timed Up and Go test (ES = −0.46). These findings provide quantitative support for clinically relevant effects on several core motor domains.

However, improvements should not be generalized to all manifestations of PD. Evidence for freezing of gait, falls, walking velocity, and some other gait parameters has been less consistent. Furthermore, Romenets et al. [26], in a randomized controlled trial comparing 24 partnered tango classes with self-directed exercise, found no between-group difference in the primary outcome of motor severity. Nevertheless, tango produced superior improvements in Mini-BESTest performance, simple Timed Up and Go, and dual-task Timed Up and Go performance.

The evidence therefore suggests that tango may be particularly relevant to dynamic balance and functional mobility, rather than producing uniform improvement across every aspect of parkinsonian motor impairment. This distinction is important clinically and reinforces the need for outcome-specific interpretation.

### 6.3. Motor–Cognitive and Complementary Psychosocial Outcomes in Parkinson’s Disease

Cognitive outcomes represent a promising but considerably less established area. Argentine tango requires movement selection, spatial navigation, attention to musical information, memory for movement possibilities, response inhibition, adaptation to the partner, and simultaneous management of multiple sensory inputs. These characteristics provide a plausible rationale for cognitive–motor benefits.

McKee and Hackney [24] reported improvement in spatial cognition following a community-based Adapted Tango intervention compared with an education control group. Conversely, Romenets et al. [26] observed only a non-significant trend toward improvement on the Montreal Cognitive Assessment, although dual-task Timed Up and Go performance improved significantly. The earlier systematic review by Lötzke et al. [8] consequently concluded that the limited number of cognitive studies prevented firm conclusions.

Evidence concerning psychosocial outcomes is similarly heterogeneous. Some studies have reported improvements in disease-related quality of life, including mobility-related dimensions, whereas others have found no significant between-group effects. Depression outcomes have also been inconsistent. Romenets et al. [26], for example, found no significant advantage for depression but reported trends toward cognitive and fatigue improvements and substantially greater enjoyment and treatment satisfaction among tango participants.

These findings are important because enjoyment and perceived value may influence adherence independently of immediate clinical effects. For chronic progressive disorders requiring sustained physical activity, an intervention that participants perceive as socially meaningful and pleasurable may offer practical advantages over repetitive exercise, although this hypothesis requires longer-term investigation.

### 6.4. Secondary Evidence Beyond Parkinson’s Disease: Healthy Aging and At-Risk Populations

The therapeutic relevance of tango extends beyond neurological disease. Aging is commonly accompanied by reductions in balance, gait adaptability, strength, cognitive–motor performance, and confidence during mobility, making multidimensional physical activities particularly relevant to healthy aging.

McKinley et al. [22] compared a community-based tango program with walking in older adults. Both activities produced beneficial changes, but tango was associated with greater clinical improvements in balance-related outcomes and walking performance, suggesting that its multidirectional and coordinative demands may provide additional challenges beyond habitual walking.

Hackney et al. [25] subsequently examined Adapted Tango in 74 community-dwelling older adults aged 59–95 years. Twenty 90-min sessions over 12 weeks improved mobility, backward and fast gait speeds, and motor–cognitive function relative to health education, with several gains maintained three months later. Importantly, general cognition did not improve significantly, again demonstrating the need to distinguish improvements in integrated cognitive–motor performance from global cognitive enhancement.

Beyond conventional healthy-aging populations, Adapted Tango has also been investigated in individuals at increased risk of cognitive decline. Wharton et al. [29] conducted a phase I randomized clinical trial in middle-aged African American women with a parental history of Alzheimer’s disease, comparing a 12-week, 20-lesson Adapted Tango intervention with a no-contact control condition. Tango participants demonstrated improvements in executive functioning and inhibitory control, as well as dynamic and static balance and functional lower-body strength. Notably, the intervention was also associated with significant reductions in several circulating inflammatory cytokines, including IL-7, IFN-γ, TNF-α, and MCP-1. Although the small sample and exploratory nature of the trial preclude conclusions regarding dementia prevention, these findings broaden the potential relevance of tango beyond established neurological disease and suggest that future studies should examine cognitive, motor, and biological outcomes concurrently.

These findings position tango as a potentially useful form of complex physical activity for older adults, particularly because it simultaneously challenges locomotion, balance, adaptation, and social engagement. Nevertheless, evidence in neurologically healthy and at-risk populations remains substantially less developed than the literature on PD, and current findings should be regarded as preliminary.

### 6.5. Psychosocial Outcomes as Complementary Evidence

Argentine tango is also relevant to well-being because participation occurs within an expressive and interpersonal context rather than solely as physical training. Music, touch, shared movement, mastery of new skills, and participation in a group may contribute to affective experience, self-efficacy, social connection, and perceived quality of life.

The broader dance literature supports beneficial psychological effects of dance and dance/movement therapy, including effects on depression, anxiety, quality of life, interpersonal functioning, and cognitive outcomes [1]. Tango-specific evidence, however, remains more limited and should not simply be inferred from dance research as a whole.

Within PD studies, indicators such as enjoyment, treatment satisfaction, quality of life, and willingness to continue dancing suggest that tango may have psychosocial value. Partnered tango may be particularly relevant because it transforms exercise into a reciprocal activity involving touch, communication, cooperation, and shared achievement. Moreover, participation in a dance community may provide opportunities to maintain social roles that are not defined primarily by illness or disability.

Nevertheless, social connectedness has rarely been treated as a primary outcome in controlled tango trials. Consequently, although qualitative observations and participant preferences support its importance, the magnitude and mechanisms of tango-specific psychosocial effects remain insufficiently quantified.

### 6.6. Limitations of the Current Evidence

Despite promising findings, the empirical literature has several important limitations. First, many studies include small samples, reducing statistical power and limiting generalizability. Second, intervention protocols vary considerably in duration, frequency, class structure, instructor expertise, partnering arrangements, and degree of adaptation. Although Berti et al. [21] identified 21 publications examining tango in idiopathic PD and found intervention reporting generally satisfactory, they also emphasized the need for more complete reporting to facilitate replication and clinical implementation.

Third, control conditions are heterogeneous, ranging from no intervention or health education to exercise, walking, Tai Chi, and alternative dance styles. This complicates determination of whether observed benefits arise specifically from tango or from physical activity, music exposure, social interaction, expectation, or structured participation more generally.

Fourth, blinding is inherently difficult in dance interventions, and participant self-selection may favor individuals already receptive to music, dance, or social activity. Attrition and adherence appear generally acceptable, but long-term maintenance remains inadequately studied. Fifth, PD dominates the literature, whereas evidence in healthy aging, other neurological populations, mental health, and community settings remains comparatively sparse.

Finally, the evidence is strongest for behavioral outcomes, particularly balance and functional mobility. Direct evidence for the neurophysiological mechanisms proposed to underlie tango—including tango-specific auditory–motor coupling, neuroplasticity, interpersonal synchrony, and affective regulation—is substantially more limited. Similarly, cognitive and psychosocial findings remain less consistent than motor outcomes.

Thus, the current evidence supports Argentine tango as a promising, feasible, and multidimensional intervention, particularly for balance and functional mobility in PD, but does not justify portraying it as a universally effective therapy. Rather, existing findings provide an empirical foundation from which specific therapeutic mechanisms and interactions can be hypothesized and tested. This distinction between demonstrated clinical outcomes and proposed integrative mechanisms is central to the Argentine Tango Therapeutic Integration Model (ATTIM) developed in the following section.

## 7. The Argentine Tango Therapeutic Integration Model

### 7.1. Rationale for the Model

The evidence reviewed in the preceding sections suggests that Argentine tango cannot be adequately conceptualized as physical exercise, rhythmic stimulation, cognitive training, or social activity in isolation. Its distinctive characteristic is the simultaneous integration of these components within a continuously adaptive, embodied interaction. Existing research has demonstrated clinically relevant effects, particularly on balance and functional mobility in Parkinson’s disease (PD), while broader dance research supports potential cognitive, affective, and neuroplastic effects. However, these domains are commonly investigated separately, making it difficult to explain how the constituent elements of tango may interact to generate therapeutic outcomes.

As described in the Methods, ATTIM was not derived from statistical modeling or formal inductive thematic analysis. It represents an author-generated conceptual integration of evidence with different levels of directness. Level I was derived from structural characteristics intrinsic to Argentine tango; Level II incorporates mechanisms supported to varying degrees by tango-specific, related dance, and indirect mechanistic evidence; Level III prioritizes outcomes reported in tango-specific clinical research; and Level IV integrates recurrent clinical and contextual factors that may modify therapeutic responsiveness. Consequently, the evidentiary support for individual pathways is not uniform, and the model should not be interpreted as demonstrating causal relationships among its components.

To address this conceptual gap, this review proposes the Argentine Tango Therapeutic Integration Model (ATTIM). ATTIM is a theoretical framework intended to organize existing evidence and generate testable hypotheses rather than a validated clinical model. It conceptualizes tango across four interconnected levels: Level I—therapeutic inputs; Level II—neuropsychological and relational mechanisms; Level III—therapeutic outcomes; and Level IV—contextual and clinical moderators. The first three levels describe the principal therapeutic architecture of the model, whereas Level IV represents a cross-cutting modulatory dimension influencing relationships within and between them. Importantly, ATTIM is neither strictly hierarchical nor unidirectional; reciprocal interactions are expected both within and between levels.

### 7.2. Level I: Therapeutic Inputs

ATTIM identifies three primary inputs that distinguish Argentine tango as a multimodal intervention: music, movement, and partner interaction.

Music provides both temporal and affective information. Rhythm establishes an external temporal structure capable of influencing movement timing through auditory–motor coupling, while melody, phrasing, accents, pauses, and changes in intensity introduce prediction, interpretation, and emotional meaning. Unlike simple rhythmic auditory stimulation, tango music requires flexible responses to a complex musical environment.

Movement constitutes the principal embodied input. Tango involves multidirectional stepping, weight transfer, changes in velocity and direction, pivots, movement initiation and termination, controlled pauses, and continuous postural adaptation. These elements challenge gait, dynamic balance, proprioception, coordination, and sensorimotor integration. Improvisation additionally transforms movement from repetitive execution into an adaptive motor-learning task.

Partner interaction represents the third input and distinguishes tango from many conventional rehabilitation approaches. Through the embrace, partners exchange tactile, proprioceptive, mechanical, and intentional information. Each dancer’s movement alters the information available to the other, creating continuous reciprocal adaptation. Partner interaction also introduces social, communicative, motivational, and affective dimensions that cannot be reproduced completely by individual rhythmic exercise.

Within ATTIM, these inputs are analytically separable but functionally interdependent: movement interprets music, partner communication modifies movement, and musical structure organizes the temporal relationship between partners.

**Figure 1:**
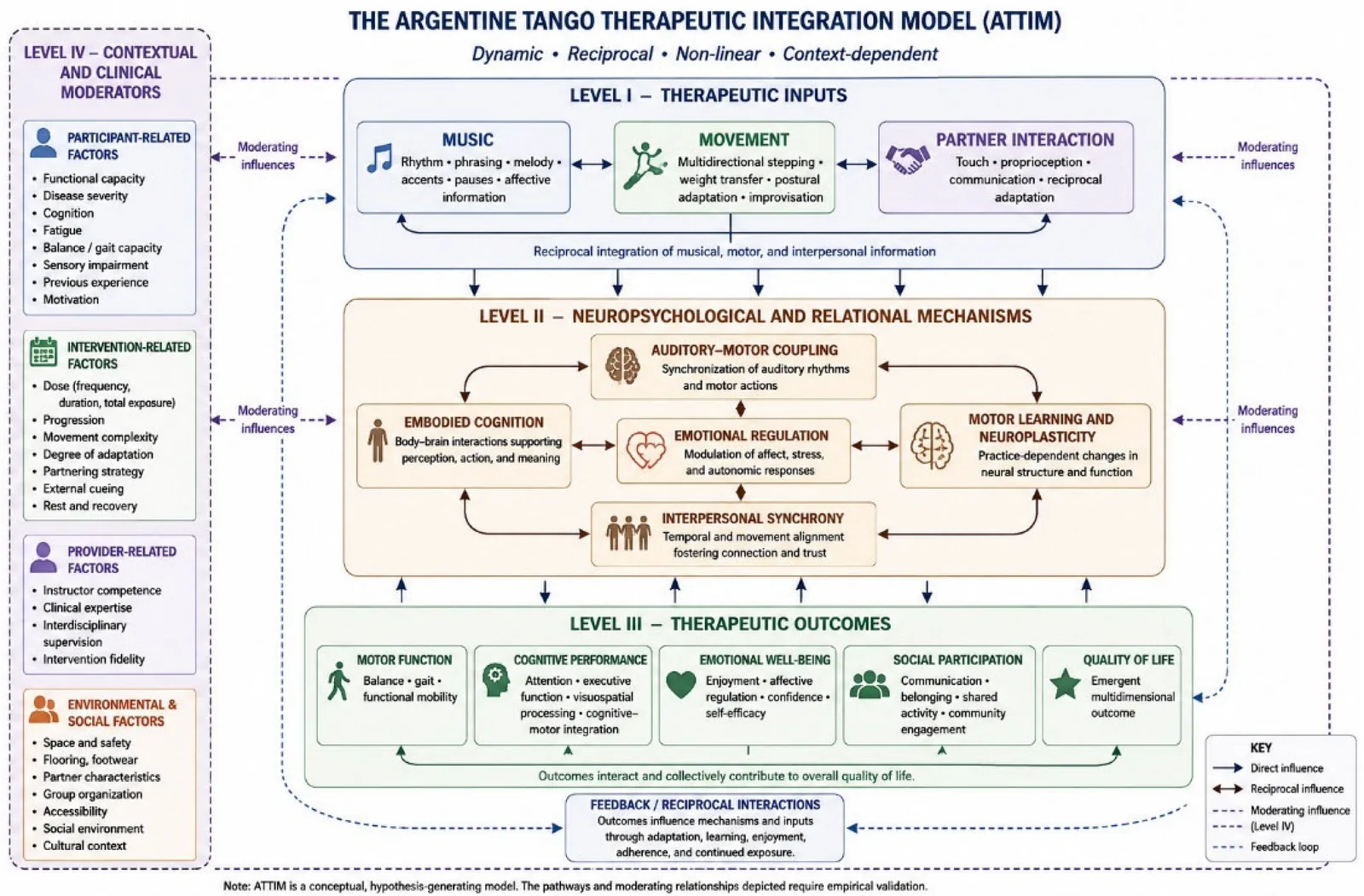
The Argentine Tango Therapeutic Integration Model (ATTIM). ATTIM conceptualizes Argentine tango as a dynamic, reciprocal, non-linear, and context-dependent therapeutic system. Level I comprises the therapeutic inputs of music, movement, and partner interaction. Level II represents proposed neuropsychological and relational mechanisms, including auditory–motor coupling, embodied cognition, emotional regulation, motor learning and neuroplasticity, and interpersonal synchrony. Level III encompasses motor function, cognitive performance, emotional well-being, social participation, and quality of life. Level IV comprises contextual and clinical moderators—including participant-, intervention-, provider-, and environment-related factors—that may strengthen, attenuate, or otherwise modify pathways within and between the first three levels. Bidirectional arrows indicate reciprocal interactions and feedback processes through which outcomes may influence subsequent engagement, adaptation, and therapeutic exposure. The evidentiary basis of the individual pathways is not uniform and ranges from direct tango-specific evidence to related evidence and indirect mechanistic support. ATTIM is proposed as an author-generated, hypothesis-generating conceptual framework; the pathways and moderating relationships represented in the model should not be interpreted as established causal relationships and require empirical validation. Colors are used solely to distinguish conceptual levels and component categories and do not indicate the strength or quality of evidence.

The four-level structure of ATTIM and the proposed reciprocal relationship among its components are summarized in Figure 1.

### 7.3. Level II: Neuropsychological and Relational Mechanisms

The second level represents processes through which therapeutic inputs may influence behavior and experience.

Auditory–motor coupling links perceived musical timing with movement planning and execution. Research on neurologic music therapy demonstrates that auditory rhythm can influence motor timing and gait through entrainment mechanisms [14]. In tango, however, coupling extends beyond synchronization with a regular pulse because dancers continually interpret variable rhythmic and melodic structures.

Embodied cognition provides a broader framework for understanding the reciprocal relationship between bodily action and cognitive processes. Tango requires decisions to be made through movement: spatial information, partner position, postural state, and musical events continuously constrain subsequent action. Cognition is therefore embedded within perception–action cycles rather than functioning independently of the body. This perspective is consistent with theoretical models emphasizing embodiment as an active factor in dance and creative arts therapies [15].

Emotional regulation may arise through the combined effects of music, movement, aesthetic experience, mastery, and interpersonal engagement. Music can evoke affect, while movement enables that affect to be expressed and modulated bodily. Enjoyment and perceived competence may further contribute to motivation and continued participation. These processes are particularly relevant in long-term rehabilitation, where sustained engagement determines cumulative therapeutic exposure.

Motor learning and neuroplasticity represent related but distinct mechanisms. Tango provides repeated practice under variable conditions, requiring prediction, error correction, sensory integration, and adaptation. Such variability may facilitate flexible motor learning rather than memorization of invariant sequences. Broader dance research provides evidence that sustained dance practice can be associated with structural and functional neural adaptations. A systematic review of randomized trials reported changes involving hippocampal volume, gray and white matter, cognitive performance, and neurotrophic factors following dance interventions [30]. Nevertheless, these findings cannot yet be attributed specifically to Argentine tango; tango-induced neuroplasticity remains a hypothesis requiring direct investigation.

Finally, interpersonal synchrony emerges through continuous reciprocal coordination between partners. Synchronization of movement has been associated more broadly with social bonding and feelings of connectedness, and contemporary neuroscience recognizes social interaction as an important but insufficiently investigated dimension of dance [20]. In tango, synchrony is particularly complex because partners coordinate complementary rather than identical actions.

### 7.4. Level III: Therapeutic Outcomes

ATTIM organizes potential therapeutic consequences into five interconnected domains: motor function, cognitive performance, emotional well-being, social participation, and quality of life.

Motor function currently has the strongest tango-specific empirical support. Improvements have been reported in balance, functional mobility, and selected gait-related outcomes, particularly in PD [8]. Within ATTIM, these effects may emerge from the convergence of rhythmic cueing, multidirectional stepping, repeated postural adaptation, motor learning, and partner-derived sensory information.

Cognitive performance includes attention, executive control, visuospatial processing, memory, and cognitive–motor integration. Tango requires continuous decision-making and simultaneous processing of music, movement, partner, and environmental information. Evidence specific to tango remains limited, although Adapted Tango has produced promising findings for spatial cognition and motor–cognitive performance [24,25]. Broader evidence suggests that dance may improve selected aspects of cognition in older adults, but effects vary across cognitive domains [31].

Emotional well-being encompasses enjoyment, affective regulation, self-efficacy, confidence, and potentially reductions in psychological distress. ATTIM proposes that these outcomes may arise not from movement alone but through interactions among music, embodied competence, interpersonal experience, and aesthetic engagement.

Social participation is both a potential outcome and a reinforcing mechanism. Tango may create opportunities for interpersonal communication, physical connection, shared learning, group belonging, and participation in meaningful activity. This may be especially important for older adults and individuals with chronic neurological conditions whose mobility limitations can restrict community participation.

Quality of life occupies the broadest outcome domain and should be interpreted as an emergent consequence rather than a direct product of any single mechanism. Improvements in mobility may increase independence; cognitive confidence may facilitate participation; enjoyment may improve adherence; and social engagement may reduce isolation. Quality of life therefore represents a potential convergence of motor, cognitive, emotional, and social effects rather than an independent endpoint.

### 7.5. Level IV: Contextual and Clinical Moderators

The therapeutic processes represented by ATTIM do not occur independently of the individual or the context in which tango is practiced. The fourth level therefore comprises contextual and clinical moderators that may influence the accessibility, intensity, safety, and effectiveness of the relationships linking therapeutic inputs, neuropsychological and relational mechanisms, and therapeutic outcomes. Importantly, Level IV should not be interpreted as a final stage occurring after therapeutic outcomes. Rather, it represents a cross-cutting modulatory level capable of influencing processes throughout the model.

Four broad categories of moderators can be distinguished. Participant-related factors include functional ability, balance capacity, gait independence, disease severity, cognitive status, fatigue, freezing of gait, cardiovascular tolerance, sensory impairment, previous movement experience, and the ability to understand and respond to instructions. These characteristics may determine whether particular movement, musical, or interpersonal demands constitute an appropriate therapeutic challenge or instead exceed the participant’s adaptive capacity.

Intervention-related factors include session frequency and duration, total therapeutic dose, progression, movement complexity, degree of adaptation, partnering strategy, external cueing, and opportunities for rest and recovery. The same therapeutic input may therefore generate different responses depending on how it is dosed and progressively modified. Current tango research remains heterogeneous in these parameters, and an optimal dose–response relationship has not yet been established [21].

Provider-related factors include instructor competence, clinical expertise, interdisciplinary supervision, and intervention fidelity. Expertise in tango pedagogy does not automatically confer competence in neurological rehabilitation, while clinical expertise alone does not ensure proficiency in tango musicality, partnering, or movement adaptation. Appropriate collaboration between rehabilitation professionals and trained tango instructors may therefore influence both safety and therapeutic specificity.

Finally, environmental and social factors include available space, flooring, footwear, partner characteristics, group organization, accessibility, and the broader social context in which the intervention occurs. These factors may modify not only physical safety but also interpersonal engagement, perceived competence, enjoyment, and continued participation.

Within ATTIM, contextual and clinical moderators may strengthen, attenuate, or alter pathways between Levels I–III. For example, increased movement complexity may enhance motor-learning demands in an appropriately challenged participant but compromise balance and engagement when task demands exceed functional capacity. Similarly, partner interaction may promote interpersonal synchrony and enjoyment when appropriately supported, yet become ineffective or unsafe when partnering demands are poorly matched to individual ability.

Level IV therefore introduces the principle of conditional therapeutic responsiveness into ATTIM: tango-based outcomes depend not only on which therapeutic components are present, but also on for whom, how, by whom, and under which conditions they are delivered. These moderator pathways remain hypothesis-generating and require prospective empirical validation.

### 7.6. Dynamic Interactions Within ATTIM

Although ATTIM is organized into four levels for conceptual clarity, its central proposition is that therapeutic processes are dynamic, reciprocal, and non-linear. Levels I–III describe the relationships among therapeutic inputs, underlying mechanisms, and potential outcomes, whereas Level IV modulates these relationships according to participant characteristics and intervention, provider, environmental, and social conditions. Consequently, a simple pathway—input → mechanism → outcome—cannot fully represent the tango experience.

For example, musical rhythm may facilitate stepping through auditory–motor coupling, improving movement fluency. More successful movement may increase perceived competence and enjoyment, which can strengthen motivation and adherence. Greater participation increases exposure to motor practice and partner interaction, potentially reinforcing both motor learning and social connectedness. Similarly, interpersonal synchrony may increase trust and enjoyment, while improved postural control may permit more sophisticated partnering and musical interpretation. Outcomes can therefore become inputs to subsequent therapeutic cycles.

ATTIM consequently proposes a system of reciprocal loops connecting biological, psychological, and social processes whose expression is continuously shaped by contextual and clinical moderators. This formulation is compatible with contemporary neuroscience perspectives that characterize dance as an unusually multidimensional activity integrating movement, music, cognition, emotion, and social interaction [20].

The model also generates empirically testable predictions. Future studies could dismantle the intervention by comparing partnered versus non-partnered tango, musical versus metronomic cueing, or improvisational versus predetermined movement; investigate whether changes in auditory–motor synchronization mediate gait improvements; examine whether interpersonal synchrony predicts adherence or psychosocial outcomes; and use neuroimaging or neurophysiological measures to test proposed plasticity mechanisms.

Thus, ATTIM should be understood not as evidence that all proposed pathways have already been demonstrated, but as a conceptual architecture for organizing existing findings and directing future research. Its principal proposition is that the therapeutic potential of Argentine tango emerges from the coordinated interaction of music, movement, and human connection through interdependent sensorimotor, cognitive, affective, and relational mechanisms, while the magnitude and expression of these effects are shaped by individual and contextual moderators.

## 8. Clinical and Practical Implications

### 8.1. Neurological Rehabilitation

Argentine tango may serve as a complementary intervention within neurological rehabilitation because it simultaneously challenges several domains commonly affected by neurological disorders: gait, dynamic balance, postural transitions, motor planning, attention, and cognitive–motor integration. The strongest clinical evidence concerns Parkinson’s disease (PD), for which systematic evidence indicates improvements particularly in balance and functional mobility [8].

From a rehabilitation perspective, tango offers task-specific opportunities to practice movement initiation and termination, multidirectional stepping, backward walking, turning, variable step length, weight transfer, and adaptation to external cues. These characteristics may complement conventional physiotherapy by embedding repeated motor practice within an enriched musical and interpersonal environment. Improvements in dual-task Timed Up and Go performance reported in PD further suggest potential relevance to cognitive–motor rehabilitation [26].

Nevertheless, tango should be regarded as an adjunct rather than a replacement for evidence-based neurological rehabilitation. Evidence outside PD remains comparatively limited, and clinical application should therefore be determined by individual goals, impairments, disease severity, cognitive status, and safety requirements.

### 8.2. Healthy Aging and Fall Prevention

Tango may also have practical value in healthy and active aging. Age-related deterioration in balance, gait adaptability, muscle function, sensory integration, and cognitive–motor performance contributes to reduced mobility and increased fall risk. Unlike repetitive locomotor exercise, tango requires continuous modification of the base of support, movement direction, velocity, and postural orientation.

McKinley et al. [22] demonstrated that community-dwelling older adults at risk of falling could participate in a structured tango program, with improvements in functional balance and mobility-related outcomes. Adapted Tango has subsequently been associated with improvements in mobility, gait, and motor–cognitive function among older adults living independently [25].

These findings support tango as a potentially useful component of multicomponent healthy-aging programs. However, improvement in balance-related measures should not automatically be interpreted as a demonstrated reduction in fall incidence. Large prospective trials examining actual falls remain necessary. Accordingly, tango is more appropriately described as targeting modifiable factors associated with fall risk than as an established stand-alone fall-prevention intervention.

### 8.3. Psychosocial and Community-Based Interventions

One practical advantage of tango is its capacity to move rehabilitation beyond a strictly clinical environment. Community-based classes may transform therapeutic exercise into meaningful social participation involving music, interpersonal communication, skill acquisition, and shared experience.

Qualitative research involving adults with PD participating in Adapted Tango has identified perceived influences on participation and quality of life [27]. Moreover, a 12-week Adapted Tango intervention involving older adults with and without PD was associated with improvement in aspects of participation, particularly social life [28]. Such findings suggest that community tango may address dimensions of functioning that are not adequately represented by impairment-based clinical measures.

Community delivery may additionally facilitate long-term engagement. Tango provides progression, aesthetic experience, and opportunities for social identity beyond the patient role. Importantly, community implementation should retain appropriate therapeutic adaptation and safety procedures rather than simply transferring participants from rehabilitation settings into conventional social tango classes.

### 8.4. Adapted Tango: Feasibility, Task Scaling, and Preservation of Tango Identity

Argentine tango can become technically demanding at advanced levels, but therapeutic tango interventions do not require participants to reproduce the complexity of advanced social or performance tango. Adapted Tango is based instead on scaling the difficulty of the task to the participant while retaining the organizational features that distinguish Argentine tango from generic exercise. Clinical implementation therefore requires a distinction between characteristics that define the identity of the dance and parameters that can be modified according to functional capacity [4,25].

Core characteristics that should be preserved include walking-based locomotion and weight transfer, responsiveness to tango music, some degree of improvisational movement selection, continuous awareness of the partner, and reciprocal communication through the dance connection. These elements constitute the functional architecture of tango even when the movement vocabulary is deliberately restricted. Accordingly, therapeutic sessions need not include technically demanding figures, rapid pivots, complex combinations, or advanced embellishments in order to retain tango-specific organization.

Task difficulty can be scaled through several parameters. Movement amplitude and velocity may be reduced; combinations can be simplified; pivots and rapid directional changes can initially be limited; additional time can be provided for weight transfer and movement initiation; and an open or otherwise modified embrace can be used when postural stability requires greater independence or visual information. External cueing, repetition, partner assistance, rest intervals, and progression rate can likewise be adjusted. Participants with greater impairment may initially practice simple forward, backward, and lateral walking, controlled initiation and termination, pauses, elementary changes in direction, and basic musical phrasing before progressing toward greater rotational, improvisational, or dual-task complexity. Progression should therefore be determined by functional ability and task tolerance rather than by chronological age or diagnostic category alone [4].

Adaptation should nevertheless have conceptual limits. If musical interpretation, active weight transfer, improvisational choice, and reciprocal partner communication are removed entirely, the activity may be more accurately described as a tango-inspired rehabilitation exercise rather than Adapted Tango. Adaptation therefore involves modifying task difficulty without eliminating the core perception–action relationships through which tango is organized. This distinction is important both clinically and methodologically because intervention fidelity determines whether findings can reasonably be attributed to tango-based practice.

Feasibility also depends on appropriate matching between participant, task, partner, and environment. Balance capacity, gait independence, freezing, fatigue, cognition, cardiovascular tolerance, sensory impairment, and previous movement experience may influence the amount and type of adaptation required. Adapted Tango should consequently be understood not as a simplified fixed choreography but as a graded therapeutic application in which tango-specific principles are preserved while motor and cognitive demands are progressively matched to individual capacity.

### 8.5. Safety, Professional Competencies, and Interdisciplinary Collaboration

Safety is a central requirement when tango is applied to clinical populations. Potential concerns include falls, fatigue, orthostatic symptoms, musculoskeletal overload, freezing episodes, and difficulties associated with turning or backward stepping. Nevertheless, appropriately structured programs have generally demonstrated acceptable adherence and safety profiles [21]. Long-term community-based Argentine tango has also been evaluated in a randomized controlled trial involving 62 individuals with PD who participated in twice-weekly classes over 12 months, with favorable changes in disease severity, balance, walking endurance, and selected gait outcomes relative to a no-intervention control group [23].

Risk management should include appropriate participant screening, sufficient space, suitable flooring and footwear, progressive task difficulty, strategically positioned rest opportunities, and adequate supervision. Individuals with substantial postural instability or cognitive impairment may require individual assessment before entering partnered group activities.

Professional boundaries are equally important. Expertise in Argentine tango does not independently confer competence in neurological rehabilitation, just as clinical expertise does not automatically provide sufficient knowledge of tango pedagogy, musicality, partnering, and movement culture. Effective clinical programs may therefore benefit from interdisciplinary collaboration among physiotherapists, occupational therapists, physicians, psychologists, music therapists, dance/movement therapists, and appropriately trained tango instructors, depending on intervention goals and population.

Hackney and Earhart [4,9] specifically emphasized methods for safely implementing PD-specific community tango programs, while subsequent dissemination research demonstrated that targeted instructor and volunteer training, standardized curricula, and attention to intervention fidelity can support community implementation.

Within the ATTIM framework, clinical practice therefore requires more than reproducing tango steps. The therapeutic value of the intervention depends on the deliberate integration of music, adaptable movement, and interpersonal interaction while respecting individual capacity, clinical objectives, professional competencies, and safety. Properly adapted, Argentine tango may provide a bridge between rehabilitation, preventive health, and meaningful community participation, while remaining complementary to established clinical care.

## 9. Discussion and Future Directions

The evidence reviewed here is not uniform in either quantity or directness. The strongest tango-specific clinical support concerns motor and functional outcomes, particularly balance and functional mobility in Parkinson’s disease. These findings are supported by multiple intervention studies and tango-specific evidence syntheses, although sample sizes and intervention protocols remain heterogeneous.

Evidence for cognitive, emotional, social, and quality-of-life outcomes is less consistent. Selected studies suggest potential benefits, but the number of tango-specific trials is smaller, outcome measures vary, and statistically significant effects are not observed uniformly. These domains should therefore be interpreted as promising but less established than motor and functional outcomes.

Several mechanisms incorporated within ATTIM remain biologically or psychologically plausible without having been demonstrated directly as mediators of tango-related clinical change. These include tango-specific auditory–motor coupling, interpersonal synchrony, emotional regulation, and neuroplastic adaptation. Evidence from rhythmic auditory stimulation, broader dance neuroscience, music-based rehabilitation, and social synchronization research supports their plausibility but cannot substitute for direct mechanistic investigation of tango.

The principal contribution of ATTIM is therefore organizational rather than confirmatory. The framework distinguishes therapeutic inputs, proposed mechanisms, outcomes, and contextual moderators while making explicit the hypothesis that their interactions may explain why tango functions as more than locomotor exercise accompanied by music. Its scientific value will depend on whether future factorial, dismantling, mediation, and moderation studies can determine which components produce which effects and under which conditions.

Despite promising evidence supporting Argentine tango as a multidimensional intervention, important methodological and mechanistic questions remain unresolved. A primary research priority is the standardization of intervention protocols. Existing studies vary in session frequency, duration, total intervention length, partnering strategies, instructor qualifications, movement content, and degree of adaptation. Although Adapted Tango is the most extensively investigated approach, heterogeneity limits replication and comparison across studies [21]. Future trials should report interventions according to standardized frameworks, including class structure, musical characteristics, progression criteria, partner roles, and instructor competencies.

Closely related is the need to establish dose–response relationships and progression principles. Current evidence does not identify an optimal frequency, session duration, or total therapeutic dose. Trials comparing different intervention doses and systematically progressing motor, cognitive, and interpersonal complexity could determine whether therapeutic effects depend primarily on exposure or on progressive challenge.

Future studies should also compare Argentine tango directly with other dance modalities, including ballroom and culturally embedded dance interventions, using comparable intervention doses, participant populations, and control of social exposure. The purpose of such studies should not be to establish a universally superior dance form, but to determine whether specific task characteristics—such as improvisational movement selection, multidirectional locomotion, partner-derived sensory information, or musical interpretation—mediate particular clinical outcomes. Component-based and dismantling designs could further distinguish effects attributable to tango-specific task architecture from those arising more generally from physical activity, rhythmic cueing, interpersonal engagement, or participation in dance. Recent evidence supports dance as an adjunct intervention in PD, but methodological quality across systematic reviews remains highly variable [32].

Long-term adherence and retention of benefits require particular attention. Most studies evaluate relatively short interventions, whereas rehabilitation and healthy aging require sustained behavioral engagement. Prospective follow-up should examine whether enjoyment, social connectedness, perceived competence, and community participation predict continued dancing and maintenance of clinical gains. Earlier tango-specific meta-analysis similarly identified long-term effects and social relationships as research priorities [8].

The cognitive domain remains insufficiently characterized. Future trials should move beyond global screening instruments and assess executive function, attention, visuospatial cognition, working memory, inhibition, and dual-task performance using standardized measures. Recent meta-analytic evidence concerning dance and cognition in PD demonstrates substantial methodological heterogeneity, reinforcing the need for domain-specific assessment [33].

A further opportunity lies in integrating biomarkers and digital technologies. Wearable inertial sensors, instrumented gait analysis, motion capture, and mobile technologies could quantify step timing, movement variability, turning, postural stability, and interpersonal coordination both during tango and everyday mobility. Digital biomarkers are increasingly capable of providing repeated, objective measurements of PD-related functioning outside laboratory environments [34]. Neuroimaging, electrophysiological methods, and neurotrophic or stress-related biomarkers could additionally investigate proposed mechanisms of neuroplasticity, although tango-specific evidence is currently lacking.

Finally, ATTIM itself requires empirical validation. Its proposed pathways and moderators should be tested rather than assumed. Factorial or dismantling designs could manipulate music, movement, and partner interaction independently; mediation analyses could determine whether auditory–motor coupling, motor learning, emotional regulation, or interpersonal synchrony explain clinical outcomes; and moderation analyses could examine whether participant characteristics, intervention dose, provider expertise, or environmental conditions alter these relationships. Longitudinal studies could further examine the reciprocal feedback loops proposed by the model.

Future research should therefore progress from asking simply whether Argentine tango works toward determining for whom it works, at what dose, under which conditions, through which mechanisms, and with which lasting effects. Such research would allow ATTIM to evolve from a hypothesis-generating conceptual framework toward an empirically supported model of integrative tango-based intervention.

## 10. Conclusions

Argentine tango represents a distinctive multidimensional practice in which musical interpretation, adaptable locomotion, improvisational decision-making, and partnered interaction are continuously integrated. Within the current evidence base, its clearest therapeutic relevance concerns Parkinson’s disease, particularly balance, functional mobility, gait-related function, and selected motor–cognitive outcomes. Systematic evidence supports favorable effects on motor severity, balance, and functional mobility, although findings remain heterogeneous across outcomes and studies [8]. Evidence concerning cognition, emotional well-being, social participation, quality of life, healthy aging, and other populations is promising but less consistent and should currently be regarded as complementary or emerging. Accordingly, Argentine tango should not be considered a replacement for established rehabilitation or formal dance/movement and music therapy, but rather a potentially complementary intervention whose therapeutic relevance may derive from the interaction of multiple components.

To conceptualize these interactions, this review proposed the Argentine Tango Therapeutic Integration Model (ATTIM), which organizes tango across four interconnected levels: therapeutic inputs; neuropsychological and relational mechanisms; therapeutic outcomes; and contextual and clinical moderators. The fourth level recognizes that therapeutic responses may vary according to participant characteristics and intervention-, provider-, environmental-, and social conditions. ATTIM conceptualizes these components as reciprocal and dynamically interacting rather than as a strictly linear therapeutic sequence, consistent with contemporary perspectives emphasizing the interconnected musical, motor, affective, and social dimensions of dance [20].

ATTIM should currently be regarded as an author-generated, hypothesis-generating conceptual framework rather than a validated therapeutic model. Future research should determine which components are therapeutically active, how they interact, for whom specific combinations are most effective, and under which conditions. Standardized interventions, comparative and dismantling designs, mediation and moderation analyses, digital movement analysis, biomarkers, and long-term trials may allow these pathways to be tested directly.

Ultimately, the therapeutic potential of Argentine tango may reside precisely in what defines it as a human practice: moving with music, adapting to another person, and creating meaningful movement together. Integrating these dimensions within rigorous interdisciplinary research may advance both tango-based rehabilitation and the broader understanding of how embodied musical and social experiences contribute to human health and well-being.

## Figures and Tables

**Table 1:** Characteristics and principal findings of tango-specific empirical studies included in the narrative synthesis.

Study	Design and Sample	Population	Tango Intervention/Dose	Comparator	Main Outcomes/Instruments	Principal Findings	Key Limitations
Hackney et al., 2007 [10]	Randomized controlled preliminary study; *N* = 19	Mild-moderate PD	20 tango classes; ~1 h, twice weekly	Group exercise	UPDRS; BBS; TUG; FOG-related measures	Both groups improved overall UPDRS; tango showed significant BBS improvement and a trend toward improved TUG.	Very small sample; short intervention; limited statistical power.
McKinley et al., 2008 [22]	Randomized comparative study; *N* = 30	Older adults at risk of falls	10 weeks; 2 h twice weekly; ~40 h total	Walking program	ABC; sit-to-stand; normal and fast walking	Both interventions produced improvements; tango showed greater clinical improvement on balance-related and walking outcomes.	Small sample; preliminary trial; findings not equivalent to demonstrated fall prevention.
Hackney & Earhart, 2009 [3]	Randomized prospective study; *N* = 58	Mild-moderate PD	20 classes; 1 h twice weekly over 13 weeks	Waltz/foxtrot; no-intervention control	BBS; 6MWT; gait and backward-walking measures	Tango and waltz/foxtrot improved balance and locomotion versus control; tango improved as much as or more than ballroom on selected outcomes.	Moderate sample; short follow-up; does not demonstrate universal superiority of tango over ballroom.
Hackney & Earhart, 2009 [11]	Prospective uncontrolled pilot; *N* = 14	Mild-moderately severe PD	10 × 90-min Argentine tango sessions in 2 weeks	None	BBS; UPDRS-III; TUG; 6MWT; gait measures	Significant improvements in BBS and UPDRS-III; selected gait changes; TUG and 6MWT changes not significant.	No comparator; very small sample; intensive short-duration protocol.
Hackney & Earhart, 2010 [9]	Randomized partnered vs non-partnered comparison; *N* = 39	Mild-moderate PD	20 × 1-h classes over 10 weeks	Partnered vs non-partnered tango	BBS; gait velocity; cadence; 6MWT; participant satisfaction	Both groups improved balance and gait, with gains retained at 1 month; partnered participants reported greater enjoyment and intention to continue.	No non-dance control; limited sample; cannot isolate effects of tango from dance practice generally.
Duncan & Earhart, 2012 [23]	RCT; *N* = 62 randomized; 52 analyzed	PD	Community-based tango; 1 h twice weekly for 12 months	No prescribed intervention	MDS-UPDRS; Mini-BESTest; FOGQ; 6MWT; gait velocity	Long-term tango was associated with improvements in disease severity, balance, gait-related function and mobility relative to control.	Attrition; unblinded participants/instructors; long-term community intervention introduces uncontrolled exposure.
McKee & Hackney, 2013 [24]	Controlled prospective clinical trial; *N* = 33	Mild-moderate PD	20 × 90-min Adapted Tango lessons over 12 weeks	Interactive health education	UPDRS-III; Brooks Spatial Task; Reverse Corsi; MoCA; FAB; FSST; TUG/dual-task TUG; PDQ-39	Tango improved spatial cognition and disease severity versus education; balance and executive measures also improved; several gains retained.	Small, unequal groups; not all outcomes showed between-group effects; multiple outcomes increase risk of chance findings.
Hackney et al., 2015 [25]	Quasi-experimental, two-group repeated-measures study; *N* = 74	Community-dwelling older adults, 59–95 years	20 × 90-min Adapted Tango sessions over 12 weeks	Health education	FSST; BBS; gait speed; motor-cognitive function; cognition; psychosocial outcomes	Tango improved mobility, backward and fast gait speed and motor-cognitive function; gains maintained at 3 months; global cognition did not significantly improve.	Non-random group assignment; unequal group sizes; substantial attrition; healthy-aging population rather than neurological disease.
Romenets et al., 2015 [26]	Randomized controlled trial; *N* = 40	Idiopathic PD	24 partnered tango classes	Self-directed exercise	MDS-UPDRS-III; Mini-BESTest; TUG/dual-task TUG; MoCA; FSS; depression; QoL; satisfaction	No between-group effect on primary MDS-UPDRS-III outcome; tango improved Mini-BESTest, TUG and dual-task TUG; cognition/fatigue showed only trends; enjoyment and satisfaction were higher.	Small sample; primary motor outcome negative; multiple secondary outcomes; limited power for cognition/fatigue.
Koch et al., 2016 [6]	Single-group pre-post feasibility study; *N* = 34	PD	Single ~90-min Tango for PD workshop	None	HSI-24; Body Self-Efficacy Scale; CEQ; aesthetic-experience measures	Immediate increases in well-being, body self-efficacy, outcome expectancy and perceived movement beauty.	Single session; no control; self-reported outcomes; selected motivated sample; no objective clinical efficacy assessment.
Holmes & Hackney, 2017 [27]	Qualitative study; *N* = 16	PD	Adapted Tango; target 20 × 90-min lessons over 12 weeks	None	Focus groups; inductive thematic analysis	Participants described perceived effects on participation, daily activities and quality of life.	Qualitative self-report; small sample; participants derived from a larger tango study; no efficacy inference.
Zafar et al., 2017 [28]	Mixed quantitative/qualitative longitudinal analysis; 88 initially enrolled	Older adults with and without PD	20 Adapted Tango lessons over 12 weeks (~30 h)	Between-population comparison; no non-tango intervention	Impact on Participation and Autonomy questionnaire; open-ended responses	Social-life participation improved over time; qualitative themes suggested resilience and persistent participation challenges.	Overlapping cohorts with earlier Adapted Tango studies; attrition; no independent non-tango control.
Wharton et al., 2021 [29]	Phase I pilot randomized clinical trial; *N* = 34	Middle-aged African American women with parental history of Alzheimer’s disease	20 Adapted Tango lessons over 12 weeks; *n* = 24	No-contact control; *n* = 10	D-KEFS executive/inhibition measures; balance and strength tests; inflammatory biomarkers	Tango associated with improved executive/inhibitory performance, balance and strength and reductions in several inflammatory cytokines.	Small, demographically specific sample; phase I exploratory study; multiple endpoints; cannot infer dementia prevention.

Abbreviations: ABC, Activities-specific Balance Confidence Scale; BBS, Berg Balance Scale; CEQ, Credibility/Expectancy Questionnaire; D-KEFS, Delis–Kaplan Executive Function System; FAB, Fullerton Advanced Balance Scale; FOGQ, Freezing of Gait Questionnaire; FSST, Four Square Step Test; FSS, Fatigue Severity Scale; HSI-24, Heidelberg State Inventory; MDS-UPDRS, Movement Disorder Society-Unified Parkinson’s Disease Rating Scale; MoCA, Montreal Cognitive Assessment; PD, Parkinson’s disease; PDQ-39, Parkinson’s Disease Questionnaire-39; QoL, quality of life; 6MWT, Six-Minute Walk Test; TUG, Timed Up and Go; UPDRS, Unified Parkinson’s Disease Rating Scale.

## Data Availability

No new data were created or analyzed in this study. Data sharing is not applicable to this article.
